# Exchange bias coupling and bipolar resistive switching at room temperature on GaSb/Mn multilayers for resistive memories applications

**DOI:** 10.1038/s41598-022-27371-9

**Published:** 2023-01-13

**Authors:** Jorge A. Calderón, Heiddy P. Quiroz, Cristian L. Terán, M. Manso-Silván, A. Dussan, Álvaro Muñoz Noval

**Affiliations:** 1 Dpto. de Física, Grupo de Materiales Nanoestructurados Y Sus Aplicaciones, Universidad Nacional de Colombia - Bogotá, Cra. 30 No. 45-03 Edificio 404 Yu Takeuchi, Lab. 121C/121B-1 Ciudad Universitaria, 110001 Bogotá, Colombia; 2 Departamento de Física Aplicada, Instituto de Ciencia de Materiales Nicolás Cabrera Y Centro de Microanálisis de Materiales, Universidad Autónoma de Madrid, 28049 Madrid, Spain; 3 Departamento de Física de Materiales. Facultad de Ciencias Físicas, Universidad Complutense de Madrid, Plaza de Ciencias, 1, 28040 Madrid, Spain

**Keywords:** Materials for devices, Nanoscale materials, Structural materials, Materials science, Nanoscience and technology, Physics

## Abstract

This work present structural, morphological, magnetic, and electrical properties of GaSb/Mn multilayer deposited via DC magnetron sputtering at room temperature and at 423 K. The samples are characterized by forming layers of 3, 6 and 12 periods of the GaSb/Mn structure. Through XRD patterns, it was possible to stablish the formation of GaSb, Mn_3_Ga, and Mn_2_Sb_2_ phases. FTIR measurements present an optical interference associated with periodicity and the homogenous thickness of the layers. HR-SEM shows the multilayer architecture with columnar microstructure in the formation of layers with grain nucleation on the surface. A ferromagnetic-like behavior was observed in the multilayers at room temperature related to the domains and interlayers interaction. Additionally, the hysteresis curves present shifts attributed to the effect of exchange bias coupling. I-V curves show RESET-SET states of the multilayer system with bipolar resistive behavior, which can be modified by external magnetic fields. The resistive switching evidenced corresponds to the conductive mechanism based on the capacitive conductance and the formation of conductive filaments in multilayer structure.

## Introduction

The formation of multilayer structures using III-V and II-VI compounds has enhanced the formation of hybrid quantum dots with interesting optical and electrical properties^1^. These studies include the control carrier absorption^1,2^, diffusion process^3^, and recombination rates of carriers^1,4^ for different applications such as infrared detectors, solar cells, photonic devices and solar cells, among others^1,5,6^. Within of these materials, the study of nanostructures based on GaSb semiconductors has gained attention due to their unique properties^7–9^, in the last decades.

According to the distribution, magnetic feature, crystalline structure and thickness of the layers, the multilayer-type architecture can exhibit different resistive properties^10^. The total resistivity is usually caused by phonon and impurity scattering, which can be associated with the background of resistivity bulk, interface scattering and grain boundary scattering. Then, according to the Matthiessen's rule, the total resistivity is given by, ρ_film_ = Δρ_phonon_ + Δρ_impurity_ + Δρ_interface_ + Δρ_grain boundary_^10,11^. This evidences that the resistivity properties can be significantly affected by the interface feature and grain size^10^. However, in the study of Resistive Random Access Memories (RRAM), resistive switching has been associated with many possible conductive mechanisms, in particular: the formation of conductive filaments or the Space Charge Limited Current mechanism (SCLC)^12^.

Nevertheless, there are other mechanisms that describe resistive changes which have recently been proposed. Among these are the negative photoconductance (NPC) effect, defined as an increase in resistance after exposure to illumination in oxide materials where the excitation, migration and compensation of the oxygen vacancy at the interface are stimulated by the incident radiation. of the material^13^. Another reported resistive switching mechanism is based on the transmission of slower mobility ions instead of electrons, which is reflected in a gradual change in current with respect to a voltage pulse^14^. In the Table 1, Resistive switching mechanism and resistive feature of different compounds are present.

**Table 1:** Resistive switching mechanism and resistive feature of different compounds.

Resistive switching mechanism	Compounds	Feature	Ref.
Proton exchange reactions	SiO_x_	Unipolar	^15^
Conducting filament	ZnO	Bipolar	^12^
Conducting filament	TiO_2_	Bipolar	^16^
Redox reaction	TiO_2_	Bipolar	^17^
Negative photoconductance effect	TiO_2_	Bipolar	^13^
Redox reaction	TaO_x_	Bipolar	^18^
Space charge limited current	Graphene oxide–Fe_3_O_4_	Bipolar	^19^
Transmission of slower mobility ions	NiO_x_	Bipolar	^14^

On the other hand, among non-volatile memories (NVM), the magnetoresistive random access memory (MRAM) is the most studied^20^. This structure consists of ferromagnetic layers separated by an ultra-thin metal oxide layer that works as a tunnel barrier. In this case, the control of the resistive state corresponds to the arrangement of the magnetization of the layer^20^. This technology is based on spin manipulation, however, the study of nanostructures that present the control of resistive and magnetic switching simultaneously, is still under study^16,21^.

Other structure types, such as crossbar structure and thin-film memristive devices, have been studied. In the first case, metallic oxides as Ti-doped NbO_2_ have been employed as a selective device for enhancing the performance of resistive non-volatile memories based on the crossbar structure, like an alternative to preventing that sneak current path problem, where the selectivity around 5 × 10^4^ with a very low off current (5 × 10^−11^ A) was reported^22^; and, for the second case, the thin film memristive devices were constructed using Al/Bi_2_WO_6_/FTO characterized by bipolar resistive switching, and, its transport properties for HRS is based on Schottky conduction mechanism, whilst, LRS is governed by Ohmic charge transport mechanism^23^. Besides, memristive devices based on Ag/GeTe/Ag and Ag/FeWO_4_/FTO have presented promising features in applications, such as memories, selectors, synaptic learning functionalities and, neurophormic computing, respectively^24,25^.

The reports from previous studies have demonstrated the formation of the multilayer systems with the formation of the interlayers due to the diffusion process between the GaSb and Mn layer, and the formation of p-type GaSb when the samples were deposited via DC magnetron sputtering^26^. However, the study of these nanostructures for applications such as random access memories based on the electrical and magnetic properties of the multilayers is still under study. This work presents a study of structural, magnetic, and electrical properties of a [GaSb/Mn]n multilayer-type architecture deposited by DC magnetron sputtering. FTIR and SEM measurements evidenced the homogeneity of the layers. The study is focused on the presence of resistive switching and magnetic behavior of the multilayers. A model of conductive mechanism is presented.

## Experimental details

[GaSb/Mn]_n_ multilayer thin films were fabricated via DC magnetron sputtering using Ga^(36.5%)^Sb^(63.5%)^ (99.995% purity) and Mn (99.9% purity) targets in argon atmosphere at 2.5 × 10^−2^ Torr of working pressure. The multilayers were deposited with alternation between a GaSb target power and a Mn target power. Initially, GaSb was deposited by applying 100 W during 20 min, then the GaSb target power was turned off, and simultaneously the Mn target power was turned on applying 60 W of target power for 15 min. This process was repeated 3, 6, and 12 times to obtain the samples and called ${[GaSb/Mn]}_{3}$, ${[GaSb/Mn]}_{6}$, and ${[GaSb/Mn]}_{12}$, respectively. These samples were deposited at room temperature (300 K) and at 423 K on Si (001), ITO, and GaSb (001) wafer substrates.

Structural characterization was carried out by X-ray diffraction (XRD). The patterns were obtained using an X’Pert Pro polycrystal diffractometer (PANalytical) with a Bragg–Brentano geometry, a Cu-Kα source and an X'Celerator detector. The software used for Rietveld refinement was X’Pert Highscore Plus. Fourier Transformation Infrared (FTIR) spectra were obtained at room temperature with a Spotlight 200 FT-IR microscopy.

Morphological properties were acquired via FEI VERIOS 460 Scanning Electron Microscope with a maximal resolution of 0.6 nm at 15 kV and high vacuum regime (~ 10^−6^ mbar). Magnetization curves were obtained using the MPMS-SQUID with EverCool system. The magnetization measurements were made at 5 K and 300 K. Finally, the Current–Voltage characteristics were measured using a Keithley 2460 Source Meter Unit (SMU—Tektronix) at room temperature and pressure conditions. The gold contact tips used to obtain the I–V curves, were positioned by an optical microscope and a precision micrometer screw. The contact area (~ 50 $\mu$m^2^) was monitored through compression of the retractable mechanism of the tips, avoiding their penetrating into the samples. The voltage values were ranged from − 5 V to 5 V within a 5-cycles measurement process. In addition, I–V measurements with fixed external magnetic field were obtained using a certificated permanent magnet with H = 7000 Oe.

## Analysis and results

**Figure 1:**
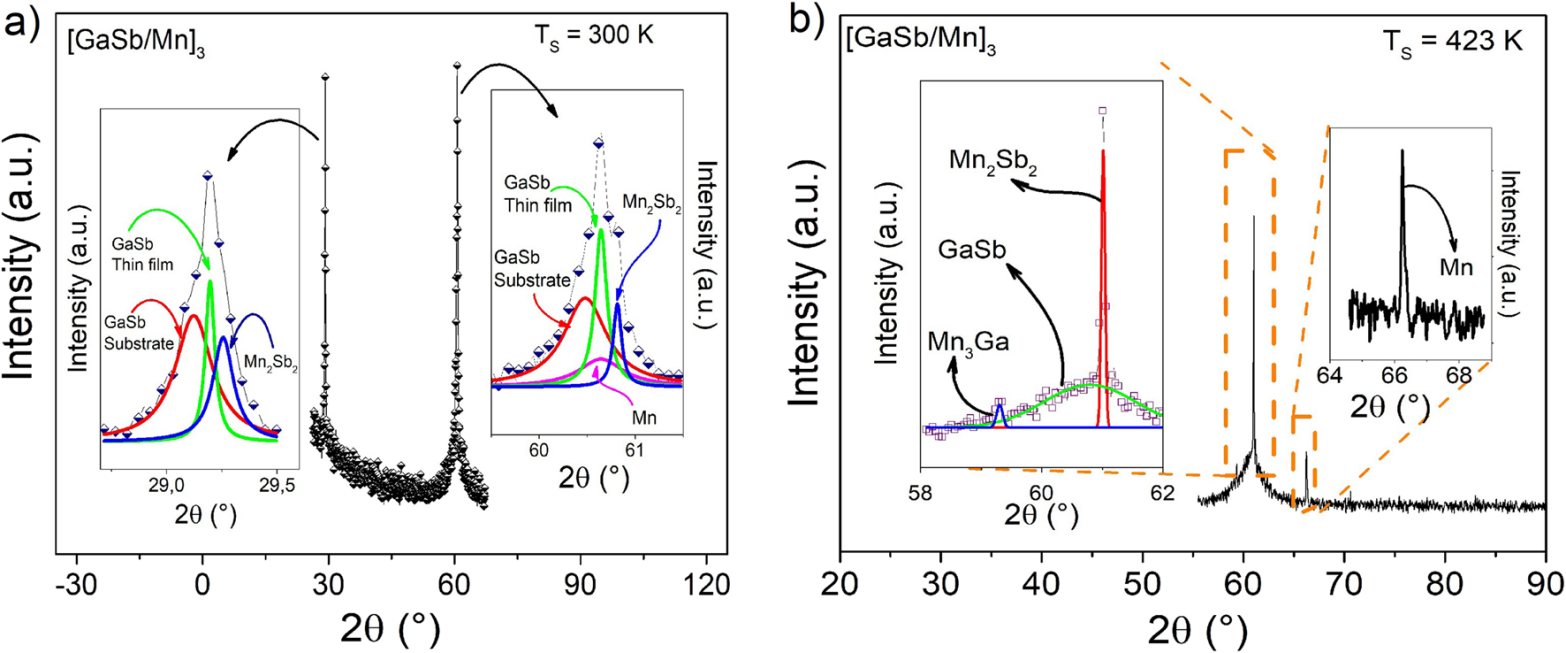
XRD patterns of ${\left[GaSb/Mn\right]}_{3}$ multilayer with substrate temperature at (**a**) 300 K and (**b**) 423 K. Insets show the deconvolution of the peaks through Lorentzian functions, which means that the binary phases have been found.

Figure 1 shows the XRD patterns of ${\left[GaSb/Mn\right]}_{3}$ architectures varying the substrate temperature (Ts). Through the Rietveld refinement, it was possible to observe the formation of Mn-α and GaSb phases according to the synthesis method; however, it was also possible to identify binary phases of Mn_2_Sb_2_ and Mn_3_Ga, which can form at the interfaces of the Mn and GaSb layers due to diffusion between them. This diffusion can be favored by the high mobility of the species involved, especially of Ga that has a melting temperature close to room temperature^7^. Therefore, in GaMnSb thin films, when the substrate temperature is increased to 423 K, the high mobility of Ga toward the substrate can occur^7^ and the formation of binary phases, such as the Mn_3_Ga phase, can be favored.

**Figure 2:**
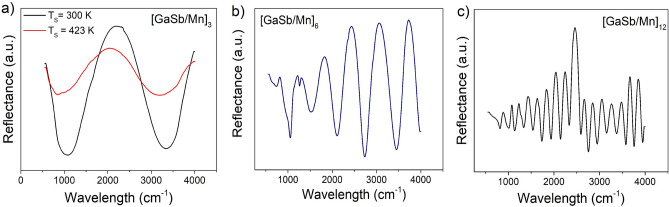
FTIR spectra of [GaSb/Mn]_n_ multilayers. Sample**s** with (**a**) $n=3$, and Ts = 300 K and 423 K; (**b**) $n=6$ and Ts = 300 K, and (**c**) $n=12$, and Ts = 300 K.

On the other hand, the FTIR spectra show interference processes evidenced by the appearance of maxima and minima modulated by a sinusoidal function associated with the distribution of the layers (Fig. 2). In the case of multilayer with $n=3$, the interference conditions allow setting the sinusoidal modulation for a higher value of wave numbers, thus presenting only two interference fringes (Fig. 2a). When the number of layer increase (n = 6 and 12), interference fringes also increase (Fig. 2b,c) due to the periodicity and the thickness of the layers. This effect is not modified by the formation of binary phases in the interfaces due to diffusion processes, which shows the homogeneity of multilayers.

When the substrate temperature is increased to 423 K, it is possible to observe that the difference between maximum and minimum is reduced and there is a shift in the inflection points of the spectrum (Fig. 2a). This may be associated with the characteristics of the interfaces due to the increase in binary phases (Fig. 1) and the diffusion processes between the elements of the layers. Although the IR vibration modes related to the binary phases are not found, the FTIR spectra could reveal their effects on interference conditions due to the interfaces features.

**Figure 3:**
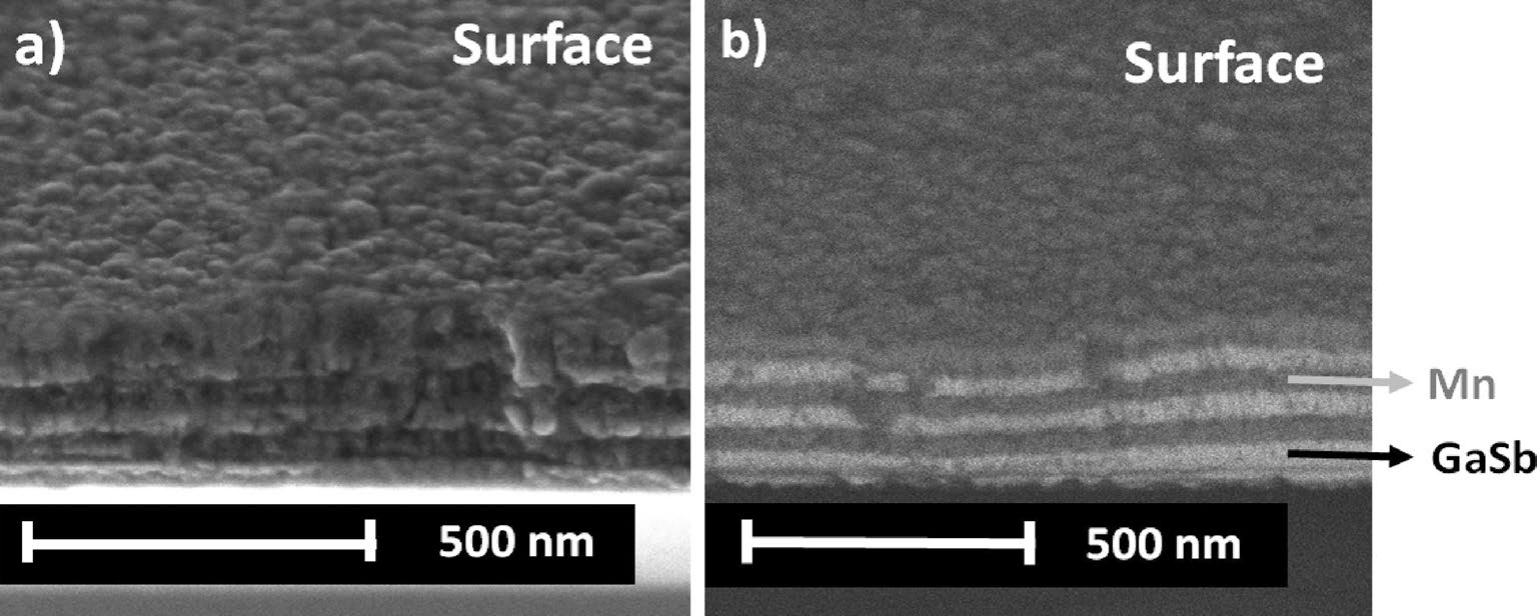
HR-SEM of [GaSb/Mn]_3_ multilayer with Ts = 300 K; (**a**) Secondary electrons (SE), and (**b**) backscattering electrons (BSE) images.

Figure 3 shows HR-SEM micrographs of the ${\left[GaSb/Mn\right]}_{3}$ multilayer. Figure 3a is a HR-SEM Secondary Electrons (SE) image whilst, Fig. 3b is Backscattering Electrons (BSE) image. The cross-section of the samples is characterized by the formation of a multilayer architecture with columnar structure (Fig. 3a) and grain size of 180.08 ± 3.24 nm on surface. In the BSE micrograph, the interface between the layers is identified (Fig. 3b), where the Mn layer corresponds to the dark gray region, and the GaSb layer to the light gray region. The morphology on the surface is governed by the high grains density, which is a prominent characteristic of the control with the deposition technique. A ${\left[GaSb/Mn\right]}_{3}$ multilayer was obtained with a thickness of 327. 27 ± 9.01 nm and GaSb and Mn layers had an average size of 56.53 ± 3.02 nm and 50.31 ± 2.13 nm, respectively.

The diffusion of the layers determined through the binary phases identified by XRD (Fig. 1) is not evident in the HR-SEM micrographs. This may be associated with diffusion effects that occur between the layers, without generating interlayers formation due to the low substrate temperature. Nevertheless, 300 K is enough temperature for the mobility of the species during the deposition process, thus favoring the formation of a columnar microstructure (Fig. 3a). This microstructure is the result of low nucleation of adatoms, similar to the first stages of growth on the substrate^27^. Columnar structures emerge when the mobility of the deposited atoms is limited, therefore, their appearance occurs throughout the bulk of the material. However, it is evident that existence of the columnar structure is favored when deposition is performed at sufficiently low temperatures.

Among others, magnetic, electrical, and surface properties of thin films are affected, sometimes strongly, by the presence of columnar structures. It was reported that the magnetic anisotropy of apparently isotropic amorphous Gd–Co films may be due to its columnar structure^28^.

**Figure 4:**
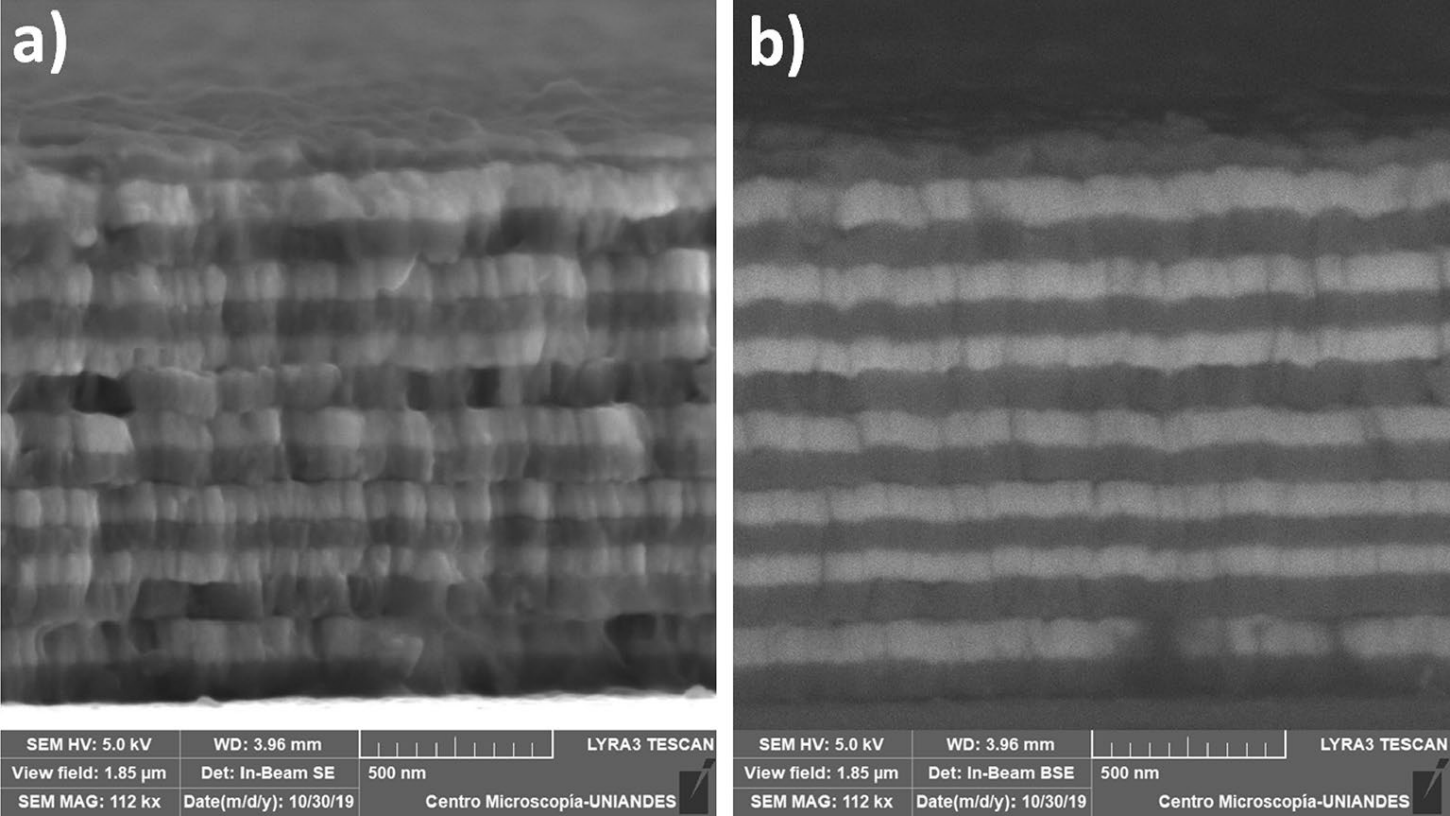
HR-SEM of a ${\left[GaSb/Mn\right]}_{12}$ multilayer at Ts = 300 K; (**a**) SE, and (**b**) BSE images. In the BSE-SEM micrograph the dark gray region corresponds to the Mn compound, while the light gray region is associated with the GaSb compound.

By increasing the number of periods in the structure from 3 to 12, it can be observed that the columnar microstructure is maintained (Fig. 4), and the layers formation is clearly defined. In this case, the thickness of the sample was 1.42 ± 0.04 µm and the grains size was 188.23 ± 5.64 nm on surface. The increase of the grain size was associated with the total deposition time (greater than that of the multilayer with n = 3), which generated increased mobility of species on the surface and greater nucleation^27^. As shown in Fig. 3, the BSE image (Fig. 4b) evidences the homogeneity of layers, where the Mn layer is represented by a dark gray region and the GaSb layer is the light gray region.

**Figure 5:**
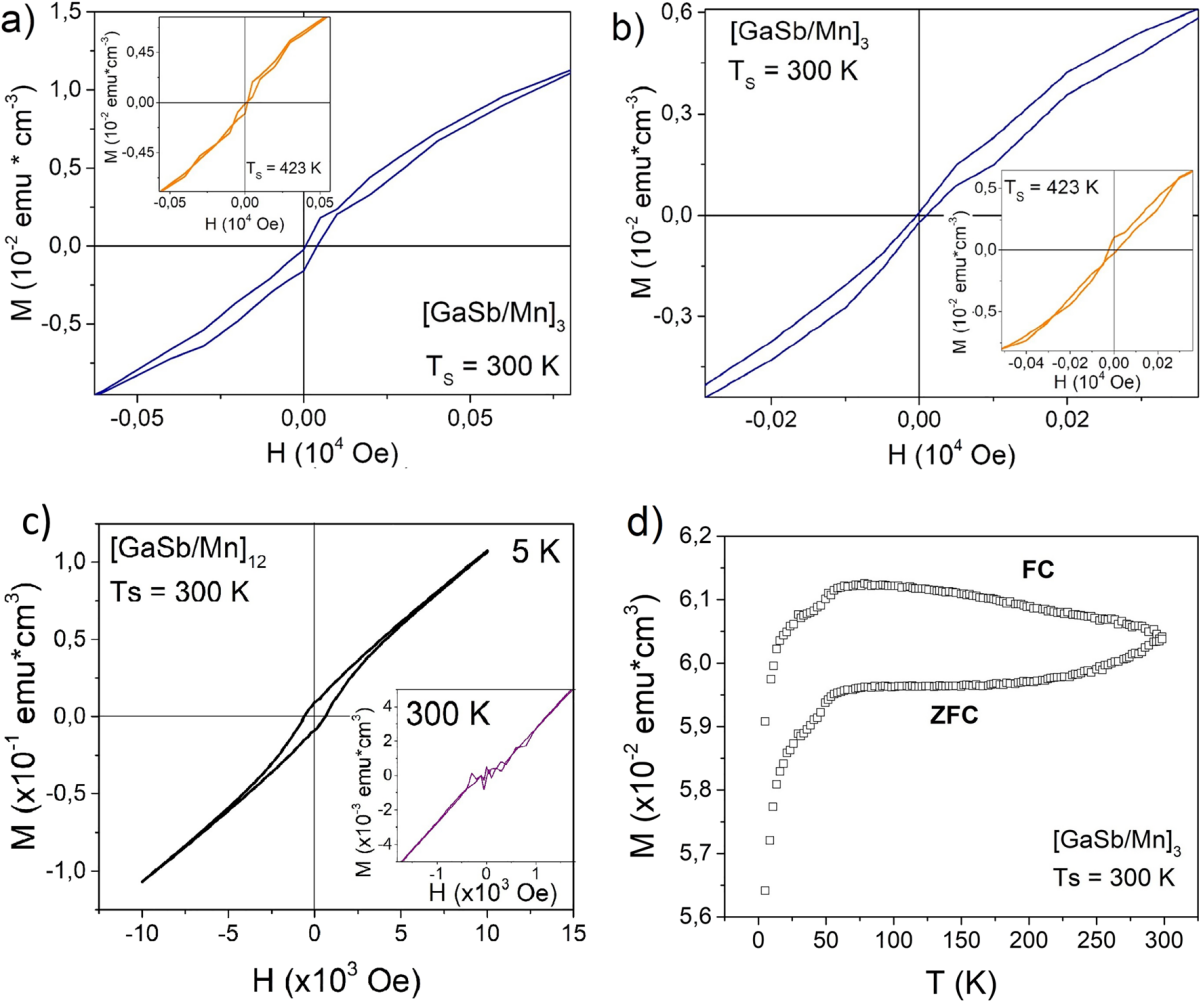
Magnetization as a function of applied magnetic field of ${\left[GaSb/Mn\right]}_{3}$ multilayers with Ts = 300 K and 423 K. The measurements were obtained at (**a**) 5 K, (**b**) 300 K, (**c**) M—H curve for the sample ${\left[GaSb/Mn\right]}_{12}$, and (**d**) ZFC—FC for 10,000 Oe measurements realized in the range from 5 to 300 K. The applied magnetic field, exchange bias, and the sample surface were parallel between them.

Hysteresis curves (Fig. 5) show the magnetization (M) as a function of the applied magnetic field (H) of the ${\left[GaSb/Mn\right]}_{3}$ multilayer varying the substrate temperature at 5 K and 300 K; and the magnetization behavior when the temperature is reduced in two cases, in the presence of a magnetic field (FC) and without it (ZFC). In both cases, the hysteresis curves present a low coercive field (Hc) without saturation. This effect was attributed to the competition between the phases identified in XRD patterns. The magnetic characteristics of all phases have been taken into account. In the case of Mn-alpha its magnetic properties correspond to antiferromagnetic material^29^, whilst, it known that GaSb is a diamagnetic compound^30^, and the ferromagnetic state of Mn_2_Sb_2_ and Mn_3_Ga phases has been reported^31–34^.

Then, the magnetization of the sample contains the contribution of magnetic characteristics of each total magnetic moment of the phases, due to the formation of the ferromagnetic phases at interfaces between GaSb semiconductor layer and Mn layer, it can be possible to obtain an environment that allows the magnetic anisotropy, and the interaction between ferromagnetic state of Mn_2_Sb_2_ and Mn_3_Ga phases produced by tiny crystals, and the antiferromagnetic moments configuration of the Mn-alpha. The opening of the hysteresis curve could be similar to ferromagnetic behavior (ferromagnetic-like behavior), but the ZFC–FC measurements presented in Fig. 5c revealed that the samples does not a critical temperature. The antiferromagnetic behavior of Mn-alpha was predominant when the temperature was reduced. In this way, the magnetization was decreasing as consequence of the phase transition of the paramagnetic state of Mn-alpha to its antiferromagnetic state.

Additionally, hysteresis curves are non-centrosymmetric when the substrate temperature was varied. This behavior is observed when the magnetic anisotropy between layers is greater than the interfacial exchange coupling, called exchange bias coupling^35,36^. This exchange bias effect can be associated with coupling between antiferromagnetic Mn and the ferromagnetic-like behavior produced through the tiny Mn_2_Sb_2_ and Mn_3_Ga crystals on the interface formation when the temperature was low (Fig. 5a)^35,36^. In this case, the sample with a T_s_ = 300 K has a positive exchange bias and its value corresponds to ~ 22.88 Oe at 5 K, while, this was ~ 3,30 Oe when the temperature of the measure was 300 K. In addition, M-H measurements were realized applying external magnetic field values between − 10,000 Oe to 10,000 Oe, and the samples presented a paramagnetic behavior when the H values were major than 1000 Oe, hence, these not have a saturation magnetization.

Also, the exchange bias at 5 K and its value at 300 K present a percentage difference of ~ 85, 54% for the sample ${\left[GaSb/Mn\right]}_{3}$. This can be related to the antiferromagnetic-paramagnetic transition phase in the Mn layers when the temperature is around 100 K. In comparison, the samples with n = 3 and n = 12 present a different exchange bias when the temperature of the measure was 5 K. In the case of the M-H curve for sample ${\left[GaSb/Mn\right]}_{12}$ shows an exchange bias value ~ 3 Oe, hence, the exchange bias was reduced when the period number $n$ was increased.

**Figure 6:**
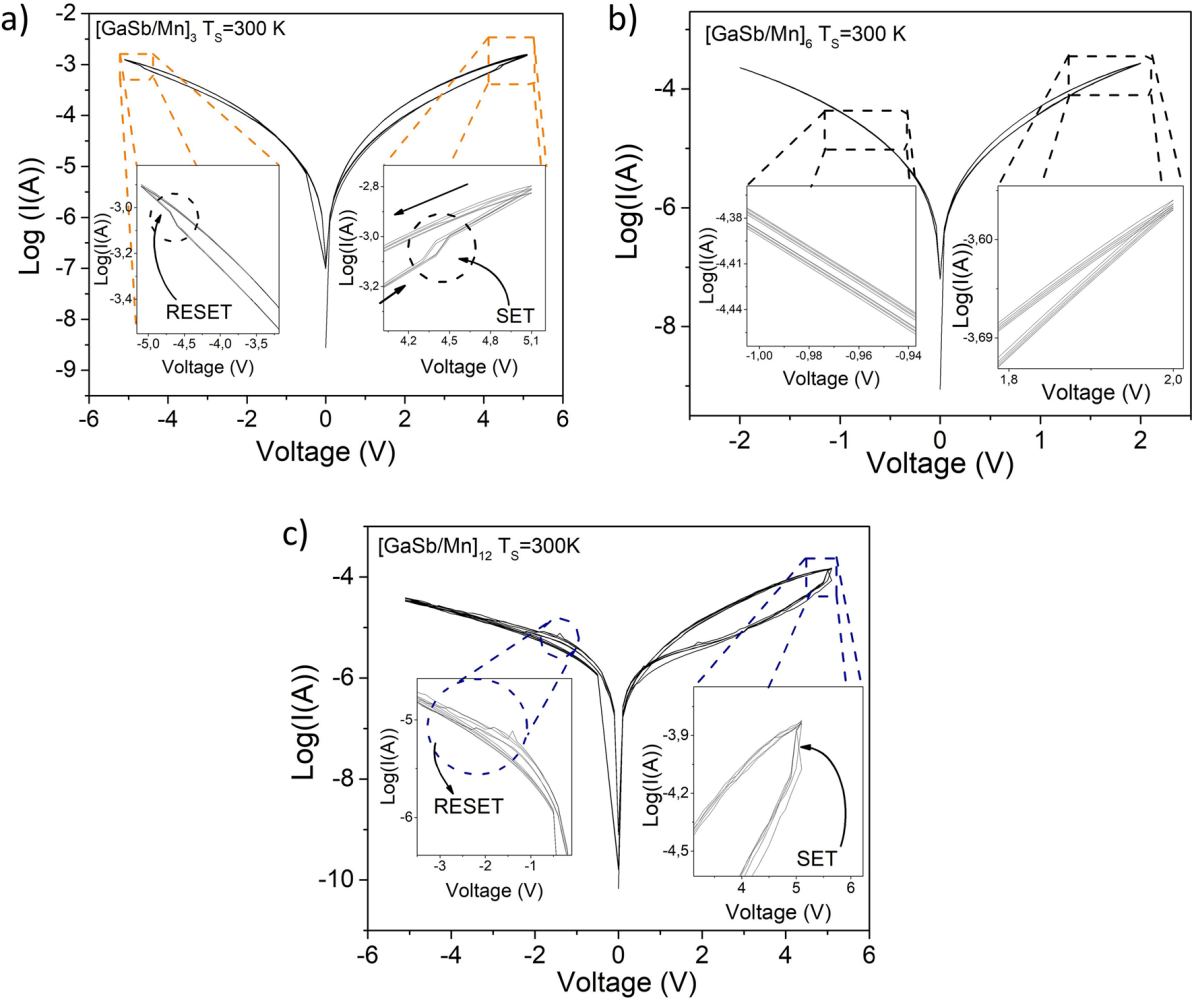
I-V curves of (**a**) ${\left[GaSb/Mn\right]}_{3}$, (**b**) ${\left[GaSb/Mn\right]}_{6}$, and **c)** ${\left[GaSb/Mn\right]}_{12}$, multilayers with Ts = 300 K. Insets shows the changes in the resistive switching.

Figure 6 presents the current–voltage (I–V) characteristics of ${\left[GaSb/Mn\right]}_{n}$ multilayers. The I–V curves can be arranged into two distinct regions as indicated by the range of the applied voltage. The first region, positive voltage region, is characterized by the change of high resistive state (HRS) to low resistive state (LRS) (right insets in Fig. 6). The second one, negative voltage region, is characterized by the change of LRS to HRS (left insets in Fig. 6). In consequence, the behavior of the ${\left[GaSb/Mn\right]}_{n}$ multilayers corresponds to bipolar switching^16^. These curves were made for 5 cycles, which shows the stability of the resistive change present in the multilayers and the potential of this architecture for applications in non-volatile memories based on the resistive change.

The resistive switching behavior in NVM has been studied through two possible conductive mechanisms: the formation of the conductive filaments^16^ or the SCLC mechanism^12^. The conductive filament model has reported to consider the ion diffusion of oxygen or metal among contacts due to the redox process, thus allowing the formation of the filament^16,37^. However, this model has been recently modified to consider the redistribution of charge carriers as responsible for the formation of the conductive filament, due to the thermo-chemical stability of the crystalline structure of semiconductors (Gibbs energy)^16^. The SCLC mechanism is observed when the contact at the junction is ohmic^19^, which allowed that carriers can readily enter the interlayer or insulator and freely flow through them. Both mechanisms were observed in the I-V curves of the ${\left[GaSb/Mn\right]}_{n}$ multilayers (see Fig. 6).

In the case of ${\left[GaSb/Mn\right]}_{n}$ multilayers, it is possible to observe the contribution of both conductive mechanisms. The conductive filament was observed in the formation of loops (insets in Fig. 6) when changes from HRS to LRS (and vice versa) occur ^16^. In addition, the switching ratio (SET/RESET) of the ${\left[GaSb/Mn\right]}_{3}$ multilayers with Ts = 300 K was 1.31, whilst, ${\left[GaSb/Mn\right]}_{12}$ multilayers was 116.73; therefore, the multilayer with 12 periods shows greater write/erase property.

On the other hand, the conductive filament is constituted by the redistribution of charge carriers within the GaSb semiconductor layer due to the presence of vacancies (gallium (V_Ga_) or antimony (V_Sb_) vacancies), the nature of the GaSb charge carrier (p-type), and Mn ions associated with the diffusion process. This conductive filament communicates the GaSb semiconductor layers with the Mn layers inside the multilayer structure. For this reason, the loop formation is affected by the voltage bias and the number of layers. Therefore, as the number of layers increases, the changes in resistivity are more difficult and the loop size increases too. The I–V hysteresis behavior revealed a major curve aperture, due to the increment of the GaSb layer number, this can be related with an increment of SCLC regions nearly to metal–semiconductor interfaces doing that the resistive switching be biggest in comparison to samples with minor GaSb/Mn spatial periods. In consequence, voltage at resistive switching occurs was increased (see Fig. 6).

**Figure 7:**
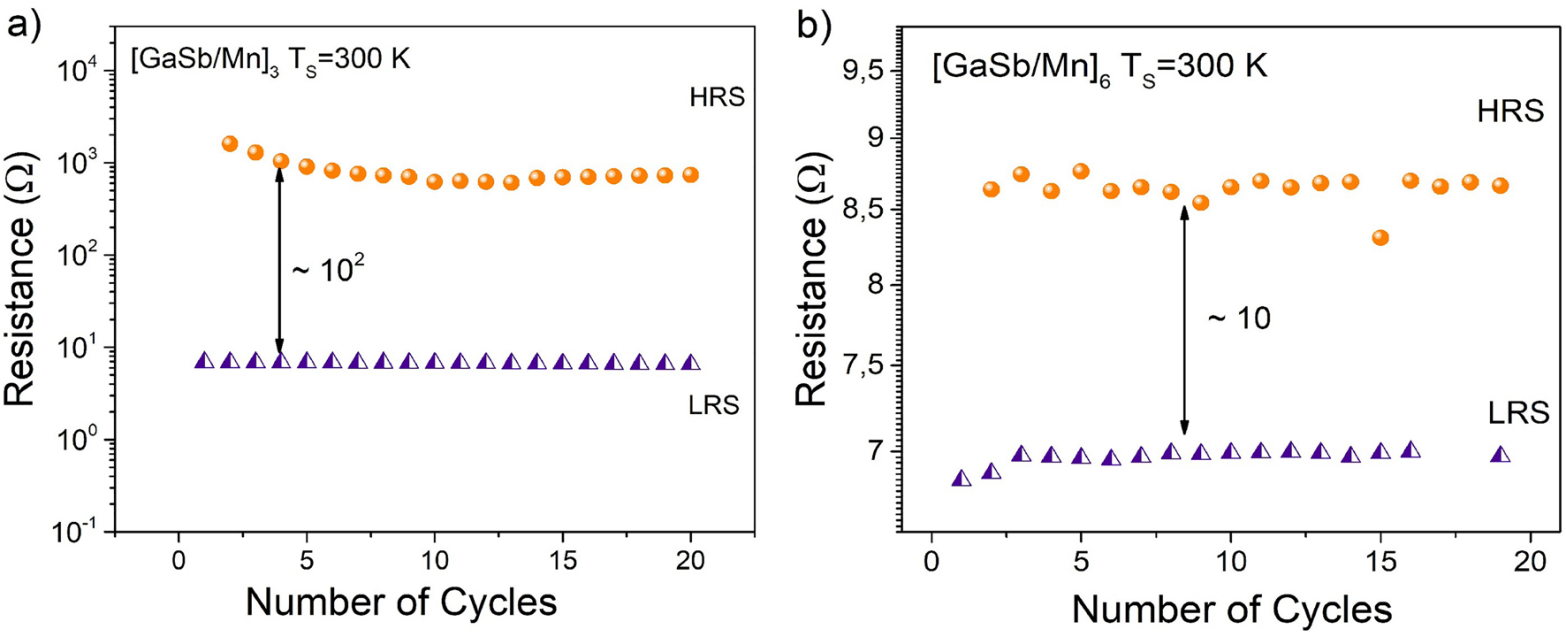
The endurance performance for devices: (**a**) ${\left[GaSb/Mn\right]}_{3}$, and (**b**) ${\left[GaSb/Mn\right]}_{6}$.

In order to evaluate the reproducibility and reliability of the multilayers, the endurance, and uniformity properties of the devices have been investigated (see Fig. 7). The resistances are read at ~ 0.5 V for the 20 cycles at room temperature. In the endurance characteristic, it is possible to observe that the HRS varied with increasing of the cycles numbers whilst LRS maintain more stable during the cycling tests. This behavior may be attributed to the stability of the conductive filaments during formation due different interface structures of the multilayers^38,39^.

The permanence and uniformity of the resistive state is modified due to the number of layers of the sample (see Fig. 7). When the layers of the multilayer are 3, it can be observed large and stable memory window around 10^2^, which is convenient for the identify the high and low resistance values. In the case of multilayer with 6 layers, the uniformity of HRS decrease and the memory widows was 10 units. This memory windows keeps stable without significant degradation up to 372 s, suggesting that the multilayer GaSb/Mn have the potential application in RRAMs.

For the construction of non-volatile memories based on resistive random access memory (RRAM) technology, thin layers of metal oxides have been used with metal–insulator-metal (MIM) structures described as two-terminal devices and in which the switching between two resistive states^40^. As an insulating material, the use of semiconductor oxides with a thin layer structure^41^, nanoparticles^40^, and more recently, nanotubes^16^ has been implemented. In the case of nanoparticles, it has been shown that the density and homogeneity of the nanoparticles affect resistive changes, as well as the possible interactions between the interfaces. Whilst, nanotube structures have shown a high directionality of the charge distribution, which strongly contributes to strong changes in resistivity and a stability of the conducting filament^16^. In comparison, multilayer structures can contribute to the formation of distributed load trapping, allowing the control of resistive changes. These multilayers show a first approach to the study of nanostructures that contribute to the construction of non-volatile memories based on two-terminal devices.

**Figure 8:**
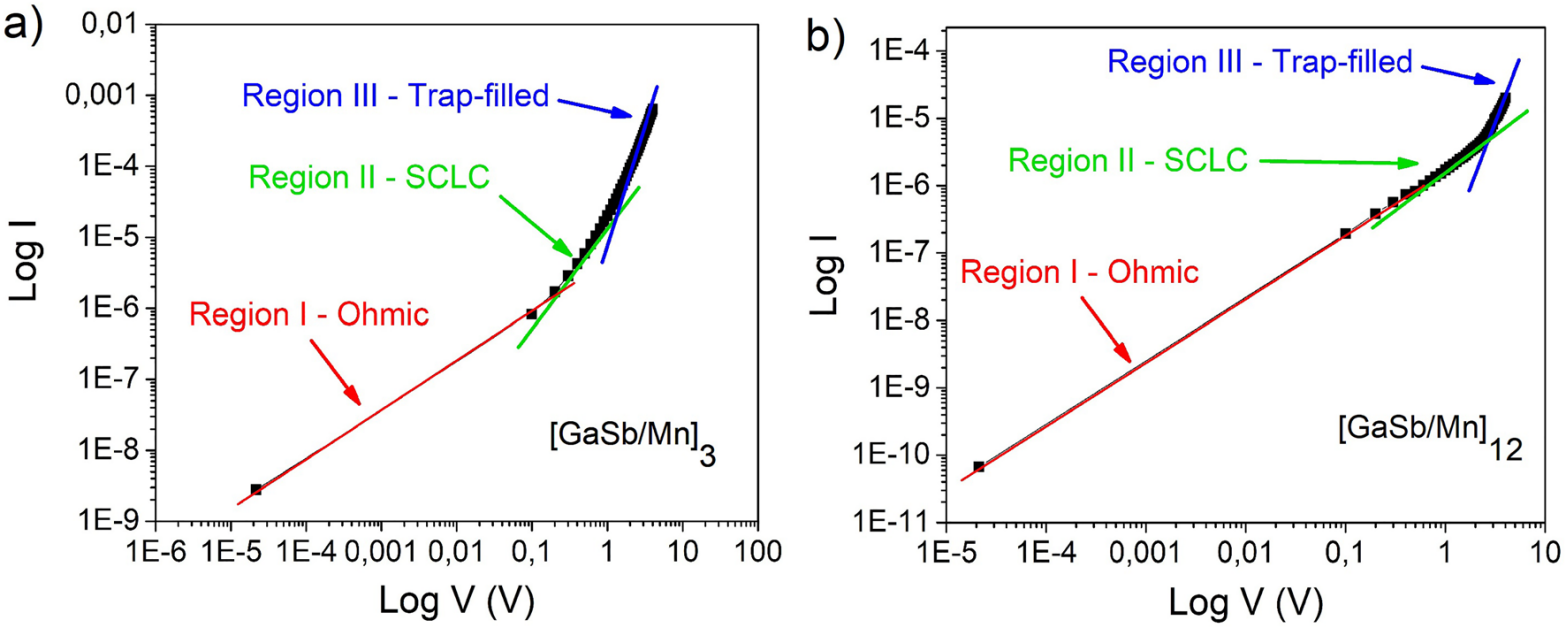
Forward bias log I—log V characteristics of ${\left[GaSb/Mn\right]}_{n}$ multilayers, and fitting results. (**a**) sample with $n=3$ and (**b**) sample with $n=12$, both at room temperature.

Nevertheless, in the low voltage region, the behavior corresponds to a SCLC mechanism. Figure 8 shows log I as a function of log V of multilayers architecture and its corresponding fitting, wherein the lowest voltage (region I—Fig. 8) presents an ohmic behavior and the region between 1 and 2 V (region II—Fig. 8) evidences the SCLC mechanism. In both cases, region I and Regions II, the fitting results were R^2^ = 0.999 and 0.993, respectively.

When the injection of the initial charge carrier is higher than that of recombination^19^, the injected carriers form a space-charge region, therefore, the current flow is limited in this region (region II). This occurs before the formation of the conductive filament. After region II, the trap-filled limited conduction mechanism is located in region III (Fig. 8). If the current is limited by the drift component of injected carriers and reorientation of the charges, the SCLC density is expressed as:

1 $$J=\frac{9\mu \varepsilon {V}^{2}}{8{L}^{3}}$$

where $\mu$ is the carrier mobility, $\varepsilon$ is the dielectric constant, $V$ is the applied voltage, and $L$ is the sample thickness^19,42^.

**Figure 9:**
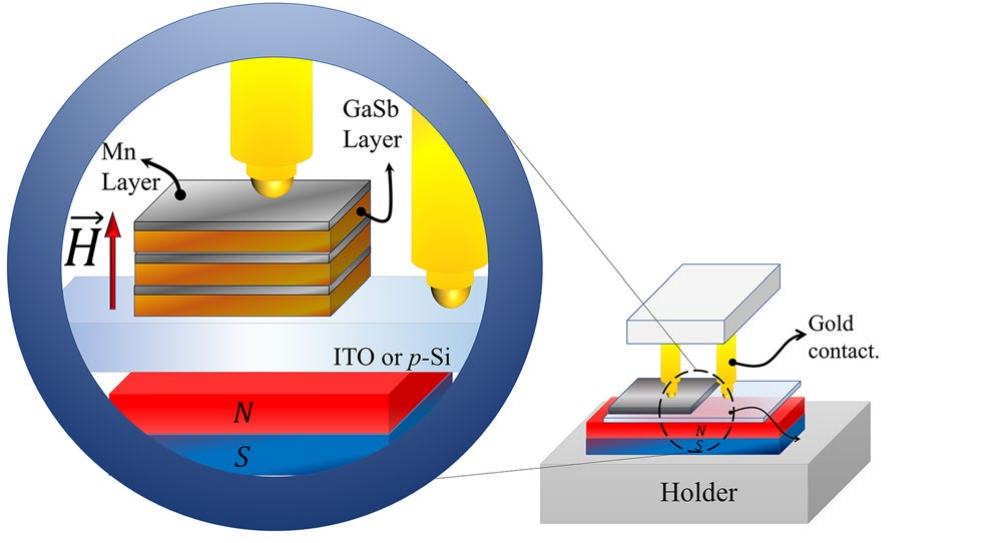
Schematic disposition of the I-V measurements with an applied an external magnetic field.

Taking into account the magnetic and resistive behavior of multilayers, Fig. 9 shows the I–V curve measurement scheme in the presence of an external magnetic field with H = 7000 Oe parallel to the current direction.

**Figure 10:**
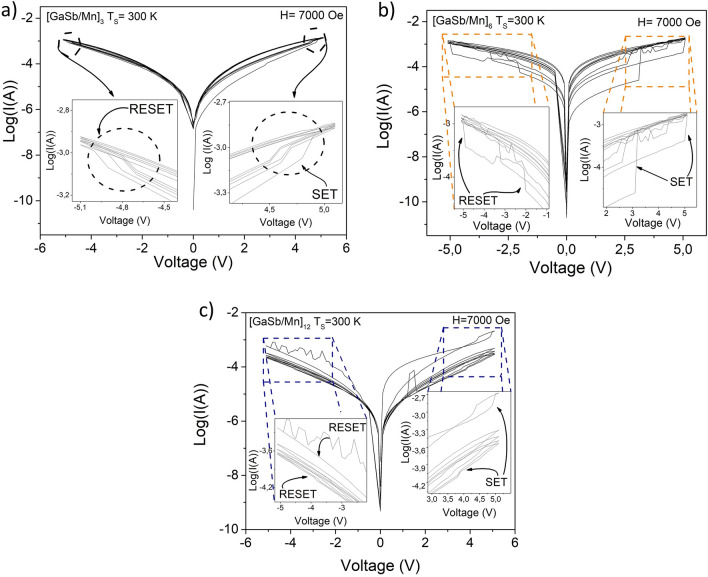
I-V curves with applied magnetic field of (**a**) ${\left[GaSb/Mn\right]}_{3}$, (**b**) ${\left[GaSb/Mn\right]}_{6}$, and (**c**) ${\left[GaSb/Mn\right]}_{12}$, multilayers with Ts = 300 K. Insets show the changes in the resistive switching.

A modification of the loops is observed in I-V curves by the magnetic field on the sample (Fig. 10), as evidenced in an increased loop area (insets in Fig. 10), thus maintaining the SET and RESET states associated with bipolar behavior. In the case that the magnetic field is applied, it is observed that the changes from HRS to LRS occur in values of approximately two orders of magnitude (from − 4 to − 6 in Log(I(A))) (Fig. 10b), while for the curves without magnetic field, HRS to LRS values of less than one order of magnitude are recorded (Fig. 6b). This behavior evidences significant a magnetic switching control on resistive properties, which makes these multilayers a promising material for Magnetic Resistive Random Access Memories (M-RRAM)^41^.

As in the I–V characteristic in the absence of a field, an increase in the size of the loop is observed when the external magnetic field is applied. This evidence a magnetoresistive effect on the multilayers that contributes to resistance changes and allows its modification through the application of an external magnetic field.

## Conclusions

This work presents the study of ${[GaSb/Mn]}_{n}$ multilayers fabricated by DC magnetron sputtering at room temperature and at 423 K. Through XRD patterns, it was possible to establish the formation of GaSb and Mn-α phases. The morphological properties evidenced the formation of a multilayer architecture with a microstructure characterized by columnar growth. From magnetic measurements, the hysteresis curves presented a low coercive field (Hc) without saturation. This effect was attributed to the competition between the phases identified in XRD patterns: Mn is antiferromagnetic, GaSb has a diamagnetic behavior, and Mn_2_Sb_2_ and Mn_3_Ga have a ferromagnetic behavior. Additionally, the non-centrosymmetric hysteresis was observed when the magnetic anisotropy between layers was greater than interfacial exchange coupling, which is associated with exchange bias coupling. From changes in the SET and RESET voltage obtained for ${\left[GaSb/Mn\right]}_{n}$ architectures, it was possible to establish the bipolar switching. For this architecture, the contribution to conduction was due to the formation of conductive the filaments and the Space Charge Limited Current mechanism. Finally, the external magnetic field modified the resistive properties, which shows a magnetoresistive effect on the multilayers.

## Data Availability

The datasets used and/or analyzed during the current study available from the corresponding author on reasonable request.
